# The complete mitochondrial genome of the *Gymnocypris selincuoensis* (Cypriniformes: Cyprinidae)

**DOI:** 10.1080/23802359.2017.1383203

**Published:** 2017-12-25

**Authors:** Jianshe Zhou, Liheng Zhang, Chi Zhang, Baohai Li

**Affiliations:** a Fisheries Research Institute, Tibet Academy of Agricultural and Animal Husbandry Sciences, Lhasa, Tibet, China;; b Henan University of Animal Husbandry and Economy, Zhengzhou, China

**Keywords:** *Gymnocypris selincuoensis*, mitochondrial genome, phylogenetic

## Abstract

The complete mitochondrial DNA sequence of *Gymnocypris selincuoensis* was determined and analyzed. This mitochondrial genome was 16,728bp in length and consisted of 37genes in the typical vertebrate mitochondrial gene arrangement. Phylogenetic analysis showed that *G. selincuoensis* is more closely related to *G. namensis* than to other species.

Lake Selincuo (88°50″–89°40″E, 31°50″–32°10″N), which is the largest lake in Tibet (Meng et al. 2012). It has an ice-covered period of more than five months annually. Only one fish species was distributed in Lake Selincuo (Chen et al. 2001).

In this study, we choose G. *selincuoensis* as our study species. The sample of *G. selincuoensis* was obtained from Lake Selincuo at an altitude of 4530 m in Tibet, China. A small portion of right pelvic fin was excised and preserved in the Fisheries Research Institute, Tibet Academy of Agricultural and Animal Husbandry Sciences. The total genomic DNA was extracted from the pelvic fin preserved in 95% alcohol. The sequence was amplified by PCR with fifteen pairs of primer.

The complete mitogenomic sequence of *G. selincuoensis* thus determined (Accession No. MF787293) had 37 genes for 13 proteins, 22 tRNAs, 2 rRNAs and a major non-coding region in a typical gene arrangement of vertebrate mitogenomes. The mitogenome base contents of *G. selincuoensis* are as follows: A 28.4%; T 27.2%; G 18.3%; C 26.1%. A low G content indicates an obvious anti guanine bias as commonly observed in other teleost fishes (Wang et al. 2016). All protein genes had an ATG start codon except for the *COX1* gene with GTG as an initiation codon. The termination codon of these 13 protein-coding genes had four types, including, ‘TA’ in *COX3*, ‘TAG’ in *ND1* and *ATP*8, ‘T’ in five genes (*ND2 ND3 ND6 COX2* and *CYTB)*, and ‘TAA’ in five genes (*ND4L*, *ND5*, *ND6*, *ATP6* and *COX1*), which was similar with other species in Cyprinidae (Wang et al. 2014)

A phylogenetic analysis (Figure 1) showed with a strong bootstrap probability (99%) that *G. selincuoensis* is more closely related to *G. namensis* than to any other species including *G. eckloni* and *G. przewalskii*. The evolutionary relationships of these analyzed species are consistent with previously reported results (He and Chen 2007). This mitochondrial genome (*G. selincuoensis*) represents the tenth mitochondrial genome to be published for Gymnocypris, and provides additional insight into evolutionary relationships within the genus.

**Figure 1.**
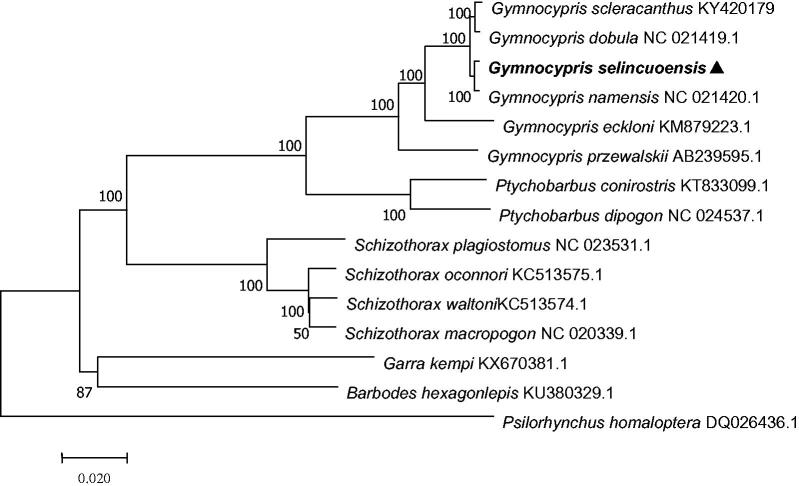
A neighbour-joining (NJ) tree of the *G. scleracanthus* was constructed using mitogenome sequences. The phylogenic tree is constructed by maximum-likelihood method with 1000 bootstrap replicates. GenBank accession numbers of mitogenomic sequences for each taxon are shown in parentheses.
